# Optimal treatment for post-MI heart failure in rats: dapagliflozin first, adding sacubitril-valsartan 2 weeks later

**DOI:** 10.3389/fcvm.2023.1181473

**Published:** 2023-06-08

**Authors:** Wenqi Tao, Xiaoyu Yang, Qing Zhang, Shuli Bi, Zhuhua Yao

**Affiliations:** ^1^ Tianjin Union Medical Center, Tianjin Medical University, Tianjin, China; ^2^ Department of Cardiology, Tianjin Union Medical Center, Tianjin, China; ^3^ The Institute of Translational Medicine, Tianjin Union Medical Center of Nankai University, Tianjin, China; ^4^ Graduate School of Peking Union Medical College, Chinese Academy of Medical Sciences, Beijing, China; ^5^ School of Medicine, Nankai University, Tianjin, China

**Keywords:** myocardial Infarction, heart failure, dapagliflozin, sacubitril-valsartan, sequential administration

## Abstract

**Background:**

Based on previous research, both dapagliflozin (DAPA) and sacubitril-valsartan (S/V) improve the prognosis of patients with heart failure (HF). Our study aims to investigate whether the early initiation of DAPA or the combination of DAPA with S/V in different orders would exert a greater protective effect on heart function than that of S/V alone in post-myocardial infarction HF (post-MI HF).

**Methods:**

Rats were randomized into six groups: (A) Sham; (B) MI; (C) MI + S/V (1st d); (D) MI + DAPA (1st d); (E) MI + S/V (1st d) + DAPA (14th d); (F) MI + DAPA (1st d) + S/V (14th d). The MI model was established in rats via surgical ligation of the left anterior descending coronary artery. Histology, Western blotting, RNA-seq, and other approaches were used to explore the optimal treatment to preserve the heart function in post-MI HF. A daily dose of 1 mg/kg DAPA and 68 mg/kg S/V was administered.

**Results:**

The results of our study revealed that DAPA or S/V substantially improved the cardiac structure and function. DAPA and S/V monotherapy resulted in comparable reduction in infarct size, fibrosis, myocardium hypertrophy, and apoptosis. The administration of DAPA followed by S/V results in a superior improvement in heart function in rats with post-MI HF than those in other treatment groups. The administration of DAPA following S/V did not result in any additional improvement in heart function as compared to S/V monotherapy in rats with post-MI HF. Our findings further suggest that the combination of DAPA and S/V should not be administered within 3 days after acute myocardial infarction (AMI), as it resulted in a considerable increase in mortality. Our RNA-Seq data revealed that DAPA treatment after AMI altered the expression of genes related to myocardial mitochondrial biogenesis and oxidative phosphorylation.

**Conclusions:**

Our study revealed no notable difference in the cardioprotective effects of singular DAPA or S/V in rats with post-MI HF. Based on our preclinical investigation, the most effective treatment strategy for post-MI HF is the administration of DAPA during the 2 weeks, followed by the addition of S/V to DAPA later. Conversely, adopting a therapeutic scheme whereby S/V was administered first, followed by later addition of DAPA, failed to further improve the cardiac function compared to S/V monotherapy.

## Introduction

Post-myocardial infarction heart failure (post-MI HF) is a common, life-threatening complication in patients with ST-elevation MI (STEMI) (1). Although early revascularization via either percutaneous coronary interventions (PCIs) or coronary artery bypass graft surgery (CABG) is widespread in clinical practice (1–4), safer and more effective medications are needed to reduce mortality and adverse outcomes in patients with HF after acute MI (AMI). The PARADISE-MI study showed that sacubitril-valsartan (S/V) was not statistically significant but yielded better effects on primary outcomes (death from cardiovascular causes and hospitalization for HF) than ramipril (hazard ratio, 0.9; 95% CI, 0.78–1.04) (5). In survivors of an AMI with left ventricular (LV) systolic dysfunction and pulmonary congestion, S/V decreased the risk of coronary outcomes (first occurrence of death from coronary heart disease, nonfatal MI, hospitalization for angina, and postrandomization coronary revascularization) compared with those for ramipril (hazard ratio, 0.86; 95% CI, 0.74–0.99) (6). The PARADISE-MI echocardiographic substudy indicated that compared to ramipril, S/V attenuated LV enlargement and improved filling pressure in patients after AMI within 1 week (7). Moreover, hypotension-related adverse events were more prevalent in the S/V group (28.3%) than the ramipril group (21.9%) (5).

Sodium-glucose co-transporter-2 inhibitors (SGLT2i), a new class of diabetes medicine, reduce hospitalization for HF (HHF) and cardiovascular (CV) mortality in patients with or without diabetes mellitus (8–10). More recently, the EMMY trial indicated that empagliflozin reduces NT-proBNP and improves heart function against the placebo in patients with HF after AMI (11).

In 2022 American Heart Association (AHA)/American College of Cardiology (ACC)/HF Society of America (HFSA) Guideline for the management of HF, SGLT2i, and S/V are classified under recommendation 1a for the treatment of HF with reduced ejection fraction (HFrEF), signifying their status as foundational therapeutic options for HFrEF (12). Combining dapagliflozin (DAPA) and S/V can substantially reduce the risk of morbidity and mortality in patients with HFrEF (13). Despite several studies, the role and impact of SGLT2i in AMI therapy have not been clearly determined. There are few studies with regard to the efficacy of S/V and DAPA alone or in combination on HF in patients with AMI. Subsequently, we conducted an investigation employing an animal model of MI in rats. Our aim was to determine whether the prompt initiation of DAPA or administering it in varying orders with S/V would yield superior outcomes in terms of heart function as compared to S/V monotherapy in rats with post-MI HF.

## Materials and methods

### Surgical animal model and experimental groups

The experimental protocol (No. 2020-C07) was approved by the Ethics Review Committee for Animal Experimentation at Tianjin Union Medical Center, China, and conformed to the Guide for the Care and Use of Research Animals established by Tianjin Union Medical Center. MI was generated in adult male Sprague-Dawley (SD) rats via surgical ligation of the left anterior descending (LAD) coronary artery. The animals were anesthetized with an intraperitoneal injection of 3% tribromoethanol (0.3 mg/kg) and ventilated using a rodent ventilator (HX-101E, TECH, China) with 70 breaths/min and a stroke volume of 420 ml/min. The chest cavity was opened in the third intercostal space, and the left coronary artery was permanently ligated by using a 6-0 silk thread. Electrocardiography (ECG) was used to demonstrate ST-elevation, which was confirmed success of the surgery. Sham-operated animals underwent the same surgical procedure except the ligation.

**Figure 1:**
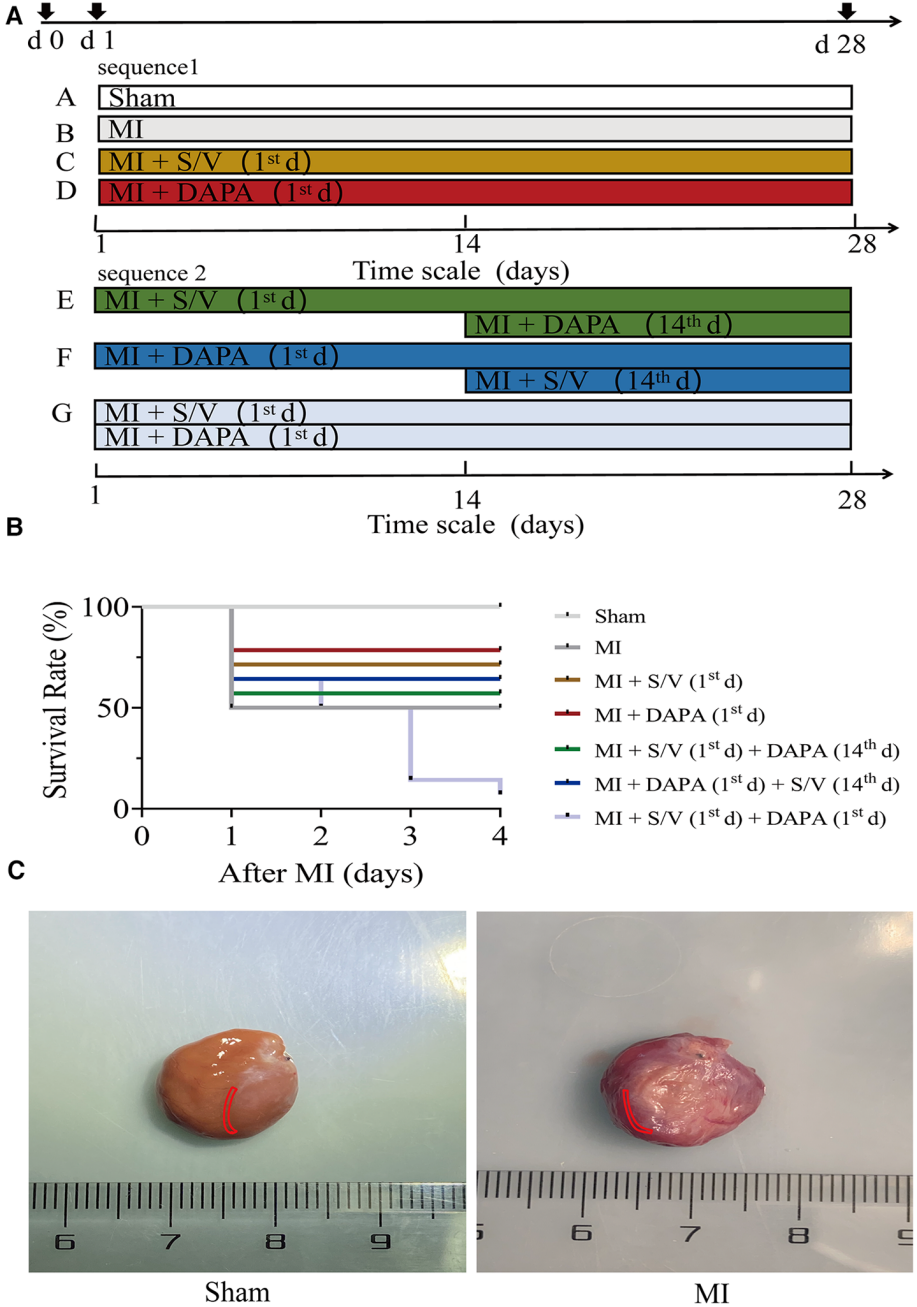
(**A**) Experimental design representative scheme, medication sequences, and duration periods; the color boxes indicate different dosing regimen: white for Sham; gray for MI; yellow for S/V (1st d); red for DAPA (1st d); green for S/V (1st d) + DAPA (14th d); blue for DAPA (1st d) + S/V (14th d); and light blue for MI + DAPA (1st d) + S/V (1st d). (**B**) Survival curves of MI 3 days after surgery, *n* = 14 per group, log-rank (Mantel–Cox) test. (**C**) In this experiment, infarct border areas were mainly used in molecular biology experiments. The Sham group was assigned to collect materials from the corresponding parts of the heart. The area is marked in the figure (in red area). S/V, sacubitril-valsartan; DAPA, dapagliflozin; MI, myocardial infarction; 1st, first day of dosing; 14th, day 14 dosing.

Animals were divided into the following seven groups: (A) Sham group: the rats were given same volume of saline by daily gavage for 28 days; (B) MI group: the rats were given same volume of saline by daily gavage for 28 days; (C) MI + S/V (1st d) group: the rats were given S/V (68 mg/kg) by daily gavage for 28 days; (D) MI + DAPA (1st d) group: the rats were given DAPA (1 mg/kg) by daily gavage for 28 days; (E) MI + S/V (1st d) + DAPA (14th d) group: S/V (68 mg/kg) treatment started on day 1, and DAPA (1 mg/kg) treatment started on day 14; (F) MI + DAPA (1st d) + S/V (14th d) group: DAPA (1 mg/kg) treatment started on day 1, and S/V (68 mg/kg) treatment started on day 14; (G) MI + DAPA (1st d) + S/V (1st d) group: the rats were given S/V (68 mg/kg) and DAPA (1 mg/kg) by daily gavage for 28 days (Figure 1A). DAPA (1 mg/ml) or S/V (68 mg/ml) was dissolved in saline. During the third day of therapy with the dual drug (group G), the death rate (91.66%) was significantly higher than that of all other groups (Figure 1B). Therefore, in subsequent experiments, we reasonably excluded this group.

### Myocardial sampling site

In this experiment, infarct border areas were mainly used in molecular biology experiments. There was a clear dividing line between the infarct border and the infarct region. The scar tissue was removed along this line. A small amount of tissue was taken along the border of the infarct to assess the damage (about 0.5 mm) (Figure 1C). The Sham group was assigned to collect materials from the corresponding parts of the heart (Figure 1C).

### ECG detection

The surface ECG (Lead II, V1) were obtained by the WorkMate Claris™ system (H700124, St. Jude Medical, United States) at 10 min, 1 day, and 28 days after operation. The rats were anesthetized with tribromoethanol during the measurements.

### Blood pressure

Systolic blood pressure (SBP) and diastolic blood pressure (DBP) were measured during day 1 and day 28 using a tail-cuff method (BP-98A, Softron, Japan), which took the average of three consecutive measurements as the mean arterial pressure.

### Echocardiography

The rats were anesthetized at day 1 or day 28 after MI with the inhalation of isoflurane. The ultrasound system (Vevo 2100, VisualSonics, Canada) was used to acquire and anatomize the images. The short-axis left atrial diameter (LA), LV end systole inner diameter (LVIDs), LV end diastolic inner diameter (LVIDd), LV end systole anterior wall thickness (LVAWs), LV end diastolic anterior wall thickness (LVAWd), LV end systole volume (LV Vols), LV end diastolic volume (LV Vold), LV fractional shortening (LVFS), and LV ejection fractions (LVEF) were assessed.

### Heart-to-tibia-length ratio

Determining the heart-to-tibia-length ratio of the rats was essential to research the effect of drugs on cardiac remodeling. After rats were sacrificed, weights of the hearts were recorded by precision balance and the tibia lengths of the rats were regularly measured.

### Measurement of infarct size

2,3,5-Triphenyltetrazolium chloride (TTC) staining was performed to evaluate the myocardial infarct size at 24 h after AMI. The hearts were sectioned into equally spaced coronal blocks (2 mm). These sections were stained with 2% TTC (G3005, Solarbio, China) at 37°C for 30 min. ImageJ software (1.8.0, National Institutes of Health, United States) was applied to measure the infarct size.

### Histological and morphological analyses

The harvested hearts were fixed in 4% paraformaldehyde and embedded in paraffin, which were cut into 5-µm thick slices. Then, the sections were stained with eosin (H&E) and Masson's trichrome. The hearts were visualized by an inverted microscope (IX53, Olympus, Japan), and the infarct and fibrosis area were measured by ImageJ (National Institutes of Health).

### Transmission electron microscope

A small piece (1 mm^3^) of the fresh left ventricle myocardium was fixed with 2.5% glutaraldehyde followed by post-fixation with 1% osmium tetroxide. After dehydration and related treatment, the ultrathin sections were observed for mitochondria under an electron microscope (HT7700 120kv, Hitachi, Japan).

### Immunohistochemistry

The heart tissue samples were fixed in 4% paraformaldehyde at room temperature (25°C) for 48 h, embedded in paraffin, and sectioned into 5-µm slices. The sections were incubated with alpha-smooth muscle actin (α-SMA) antibody (19,245, Cell Signaling Technology, 1:400) at 4°C overnight and incubated with secondary antibody for 1 h at room temperature. Sections were incubated with 3, 3′-diaminobenzidine tetrahydrochloride (DAB) substrate (SW1020, Solarbio, China) and then counterstained with hematoxylin. The image was also captured by Olympus inverted microscope and the number of α-SMA-positive cells was analyzed by ImageJ (National Institutes of Health).

### Terminal deoxynucleotidyl transferase-mediated dUTP nick-end labeling staining

Post routine dewaxing with xylene and dehydrating with gradient ethanol, these sections were detected for apoptosis levels using terminal deoxynucleotidyl transferase-mediated dUTP nick-end labeling (TUNEL) staining, following the manufacturer's instructions (11684817910, Roche, Germany).

### Western blot analysis

LV specimens from the infarct border area were homogenized in RIPA buffer (CW2333, CWBIO, China) with protease (CW2200, CWBIO, China) and PhosSTOP (04906845001, Roche, Germany) inhibitors. The Sham group was assigned to collect materials from the corresponding parts of the heart. The samples were denatured, separated by SDS-PAGE electrophoresis and transferred to a polyvinylidene difluoride (PVDF) membrane. Protein extracts (50 µg) were subjected to Western blot analysis using the following antibodies: mouse anti-GAPDH (60004-1-Ig, Proteintech, 1:5,000), rabbit anti-Bax (14,796, Cell Signaling Technology, 1:1,000), rabbit anti-Bak (12,105, Cell Signaling Technology, 1:1,000), rabbit anti-Cytochrome c (Cytc) (11,940, Cell Signaling Technology, 1:1,000), rabbit anti-Caspase-9 (ab184786, Abcam, 1:1,000), rabbit anti-Cleaved Caspase-9 (9,507, Cell Signaling Technology, 1:1,000), rabbit anti-Caspase-3 (14,220, Cell Signaling Technology, 1:1,000), rabbit anti-Cleaved Caspase-3 (9,664, Cell Signaling Technology, 1:500), rabbit anti-SOD2 (13,141, Cell Signaling Technology, 1:1,000), rabbit anti-SOD2/MnSOD2 (acetyl-SDO2) (ab137037, Abcam, 1:1,000), rabbit anti-α-SMA (19,245, Cell Signaling Technology, 1:1,000), rabbit anti-TGF-β (3,711, Cell Signaling Technology, 1:1,000), rabbit anti-Smad2 (5,339, Cell Signaling Technology, 1:1,000), rabbit anti-Phosho-SMAD2 (p-SMAD2) (18,338, Cell Signaling Technology, 1:1,000), rabbit anti-COL1A1 (91,144, Cell Signaling Technology, 1:1,000), rabbit anti-COL3A1 (ab7778, Abcam, 1:1,000). Secondary antibody was horseradish peroxidase–conjugated anti-rabbit (W401B, Promega, 1:5,000), and anti-mouse (W402B, Promega, 1:5,000). Bands were visualized using enhanced chemiluminescent ECL. Immunoblots were quantified by using ImageJ (National Institutes of Health).

### Real-time PCR

Total RNA was isolated from cardiomyocyte using the commercial RNApure Tissue&Cell Kit (CW0584, CWBIO, China), according to the manufacturer's instructions. First-strand cDNA was synthesized using a TransScript® All-in-One First-Strand cDNA Synthesis SuperMix for qPCR (One-Step gDNA Removal) (AT341-02, TransGen Bio, China). The primers used are shown in Table 1.

**Table 1:** Sequences of the primers in qPCR.

Gene	Primers
Bcl2	FP: TGTGGATGACTGAGTACCTGAACC
	RP: GGTCGCATGCTGGGGCCATATAGT
Bax	FP: AGGCGAATTGGCGATGAACTGG
	RP: CTAGCAAAGTAGAAAAGGGCAACC
Bak	FP: ATGGCATCCGGACAAGGACCA
	RP: CTTGTTCCTGCTGGTGGAGGTAA
Cytc	FP: ATCAGGGTATCCTCTCCCCA
	RP: AGGAGGCAAGCATAAGACTGGA
Caspase-9	FP: ACACCAGAAACACCCAGGC
	RP: GTATGCCATATCTGCATGTCTCT
Caspase-3	FP: GTGGAACTGACGATGATATGGC
	RP: CGCAAAGTGACTGGATGAACC
SOD2	FP: GAGGTCCTGCAGTGGTACAG
	RP: GTGAACAATCTGAACGTCACCG
α-SMA	FP: GCCGCTGAACCCTAAGGCCAAC
	RP: TGAGTCACGCCATCTCCAGAGT
TGF-β	FP: GAGAGCGCTGACCCGGA
	RP: CCCGAATGTCTGACGTATTGAAG
COL3A1	FP: ATTTTGGCACAGCAGTCCAAT
	RP: ACAGATCCCGAGTCGCAGA
COL1A1	FP: CCTCCTGACGCATGGCCAAGAA
	RP: ATAGCACGCCATCGCACACAG
Nppb	FP: CAGCAGCTTCTGCATCGTGGAT
	RP: TTCGGTACCGGAAGCTGTTGCA
GADPH	FP: TGATGGCAACAATGTCCACTTT
	RP: TAGAGACAGCCGCATCTTCTTG

α-SMA, alpha-smooth muscle actin.

### ELISA

Serum of growth stimulation expressed gene 2 (ST2) (ab255716, Abcam, England), NT-proBNP (NBP2-68140, Novus Biologicals, United States), and cTnI (ab246529, Abcam, England) were determined according to the manufacturers’ protocols for the respective kits. The level of cTnI in serum was measured at 24 h after AMI. The level of ST2 and NT-proBNP in serum was measured at 28 d after AMI.

### mRNA library construction and RNA-seq analysis of gene expression

Total RNA extractions of the left ventricles from cardiac tissues of sham group, MI group, and DAPA group (three biological replicates per group) were performed and subjected to RNA-sequencing with HiSeq X ten PE150NovaSeq 6,000 in Shanghai GeneChem Company. Differential expression analysis of two groups was performed using the DESeq2 R package (14). Genes with a *p*-value <0.05 and |log_2_FoldChange| > 1 found by DESeq2 were assigned as differentially expressed. We used the jvenn library to figure out differentially expressed genes (DEGs) overlapping between each two groups (15). DEGs classified by Gene Ontology (GO) enrichment analysis, Reactome pathway enrichment analysis, and Bayesian network plots for enrichment analysis were applied by the clusterProfiler R package, ReactomePA R package, and CBNplot R package (16–18). Enrichment analysis results were visualized using the ggplot2 package as well as the pheatmap package in R software.

### Statistical analyses

All data in this study were used to determine the means and SDs. Student's *t* test was used to assess the differences between two groups. Multigroup comparisons (more than two groups) were performed by one-way analysis of ANOVA, followed by Tukey's multiple-group comparison test or Bonferroni's multiple-group comparison test. The Kaplan–Meier method with a log-rank test was used for survival analysis. Data were analyzed using GraphPad (Prism 8, GraphPad Software, United States) and Images were processed using Adobe Illustrator (2019, Adobe, United States).

## Results

### HF model was induced by ligation of LAD coronary artery

**Figure 2:**
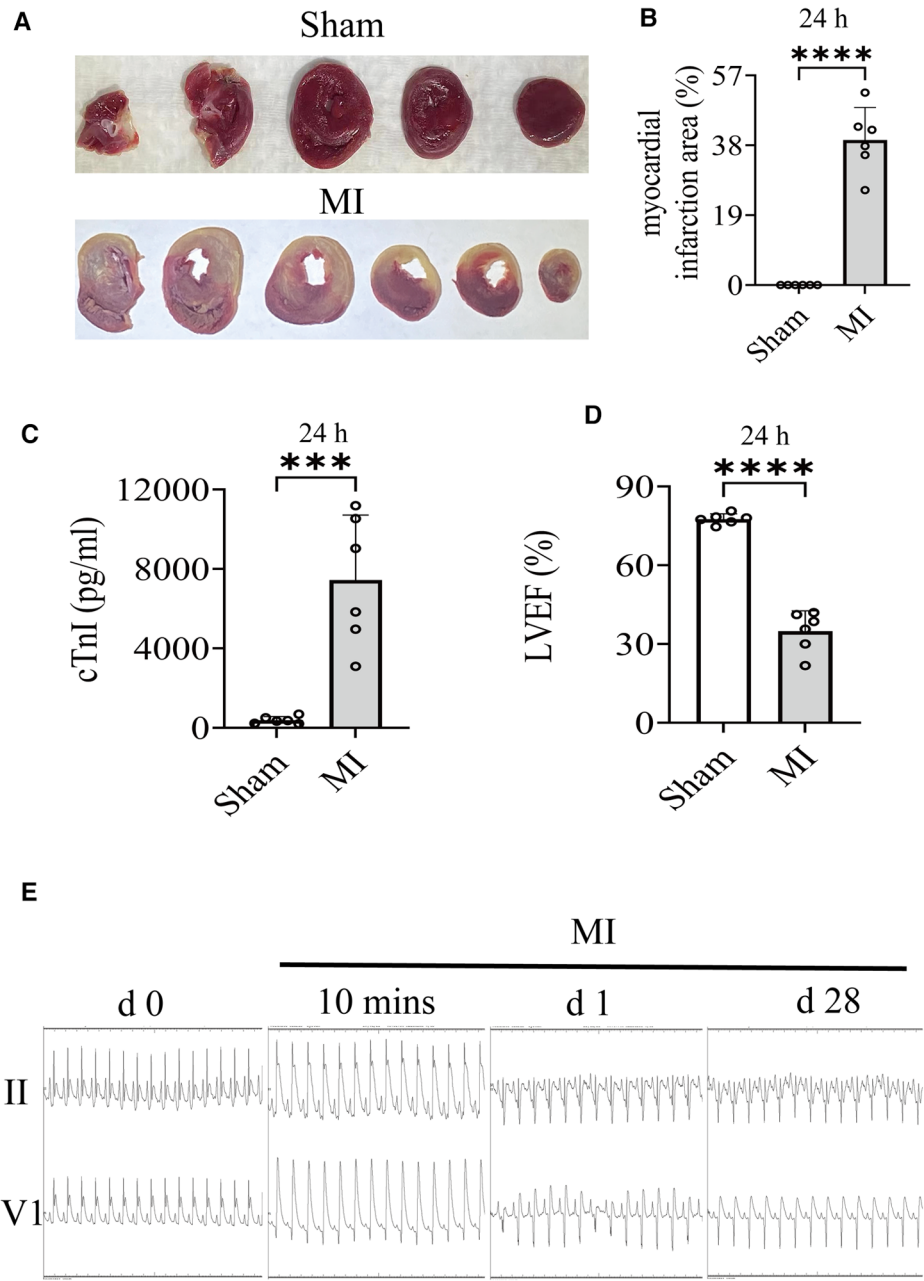
(**A,B**) Images of infarct area determined by TTC stain (24 h) and comparisons of indicated parameters. (**C**) On the first postoperative day (24 h), the level of cTnI in serum was measured. (**D**) On the first postoperative day (24 h), LVEF of rat was quantified by echocardiography. (**E**) Electrocardiogram (Lead II, V1) changes, including QRS, ST-segment, and T-wave changes, have been recorded in the groups. Student's *t* test, *n* = 6 per group. ***p < 0.001; ****p < 0.0001. LVEF, left ventricular ejection fractions; MI, myocardial infarction; TTC, 2,3,5-Triphenyltetrazolium chloride.

The results of TTC staining showed that the infarct size of the MI group was 39.43% ± 8.84% (Figures 2A,B). As shown in Figure 2C, cTnI in the MI group was substantially higher than that in the Sham operation group. The dynamic ST-segment elevation of the leads (II, V1) and T-wave height, combining with QRS, were observed during the procedure (Figure 2E). On the first postoperative day, ECG showed significant ST segment depression in leads II and appearance of abnormal Q wave in leads V1 within 24 h, which continued until the final sacrifice (Figure 2E). Simultaneously, LVIDs and LV Vols were increased from 1 day after MI surgery (Table 2). The LVAWs, LVFS, and LVEF have significantly changed from 1 day (Table 2 and Figure 2D). Moreover, the LVIDd, LVAWd, and LV Vold of rats were similar between the MI and sham groups at 1 day, suggesting that the cardiac has not yet been remodeled (Table 2).

**Table 2:** Echocardiography parameters 1 day after MI surgery.

	Day 1	
	Sham	MI
LA (mm)	4.02 ± 0.41	4.78 ± 1.04
LVIDs (mm)	3.33 ± 0.27	5.31 ± 0.50^a^
LVIDd (mm)	6.31 ± 0.56	6.42 ± 0.64
LVAWs (mm)	3.29 ± 0.22	2.04 ± 0.31^a^
LVAWd (mm)	1.96 ± 0.12	2.06 ± 0.42
LV Vols (µl)	45.41 ± 8.82	137.21 ± 28.58^a^
LV Vold (µl)	203.84 ± 41.44	212.26 ± 46.21
LVFS (%)	47.22 ± 2.05	17.18 ± 4.20^a^

LA, left atrial diameter; LVIDs, left ventricular end systole inner diameter; LVIDd, left ventricular end diastolic inner diameter; LVAWs, left ventricular end systole anterior wall thickness; LVAWd, left ventricular end diastolic anterior wall thickness; LV Vols, left ventricular end systole volume; LV Vold, left ventricular end diastolic volume; LVFS, left ventricular fractional shortening; LVEF, left ventricular ejection fractions; MI, myocardial infarction.

Data are presented as the mean ± SEM, Student's *t* test, *n* = 6 per group.

^a^ Compared with Sham group, *p* < 0.05.

### DAPA and/or S/V therapy could attenuate myocardial remodeling and preserve cardiac function after experimental MI in rats

**Figure 3:**
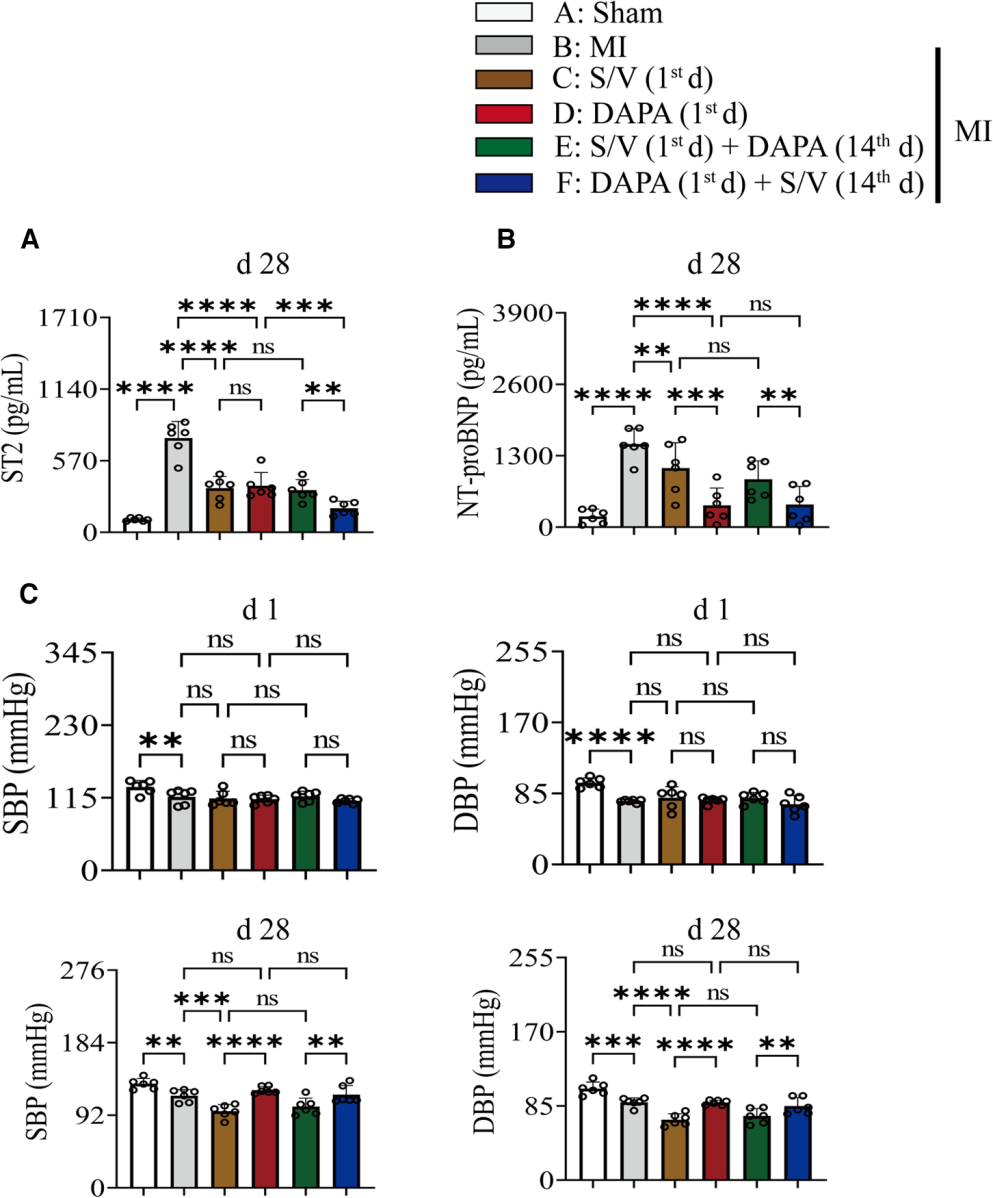
(**A**) On day 28, the level of ST2 in serum was measured. (**B**) On day 28, the level of NT-proBNP in serum was measured. (**C**) Rats SBP and DBP were measured at baseline and after 4 weeks of the postoperative day. One-way ANOVA with Tukey's *post-hoc* test, *n* = 6 per group. *p < 0.05; **p < 0.01; ***p < 0.001; ****p < 0.0001; ns, no significance. A, Sham; B, MI; C, S/V (1st d); D, DAPA (1st d); E, S/V (1st d) + DAPA (14th d); F, DAPA (1st d) + S/V (14th d). S/V, sacubitril-valsartan; DAPA, dapagliflozin; MI, myocardial infarction; 1st, first day of dosing; 14th, day 14 dosing; SBP, systolic blood pressure; DBP, diastolic blood pressure.

**Figure 4:**
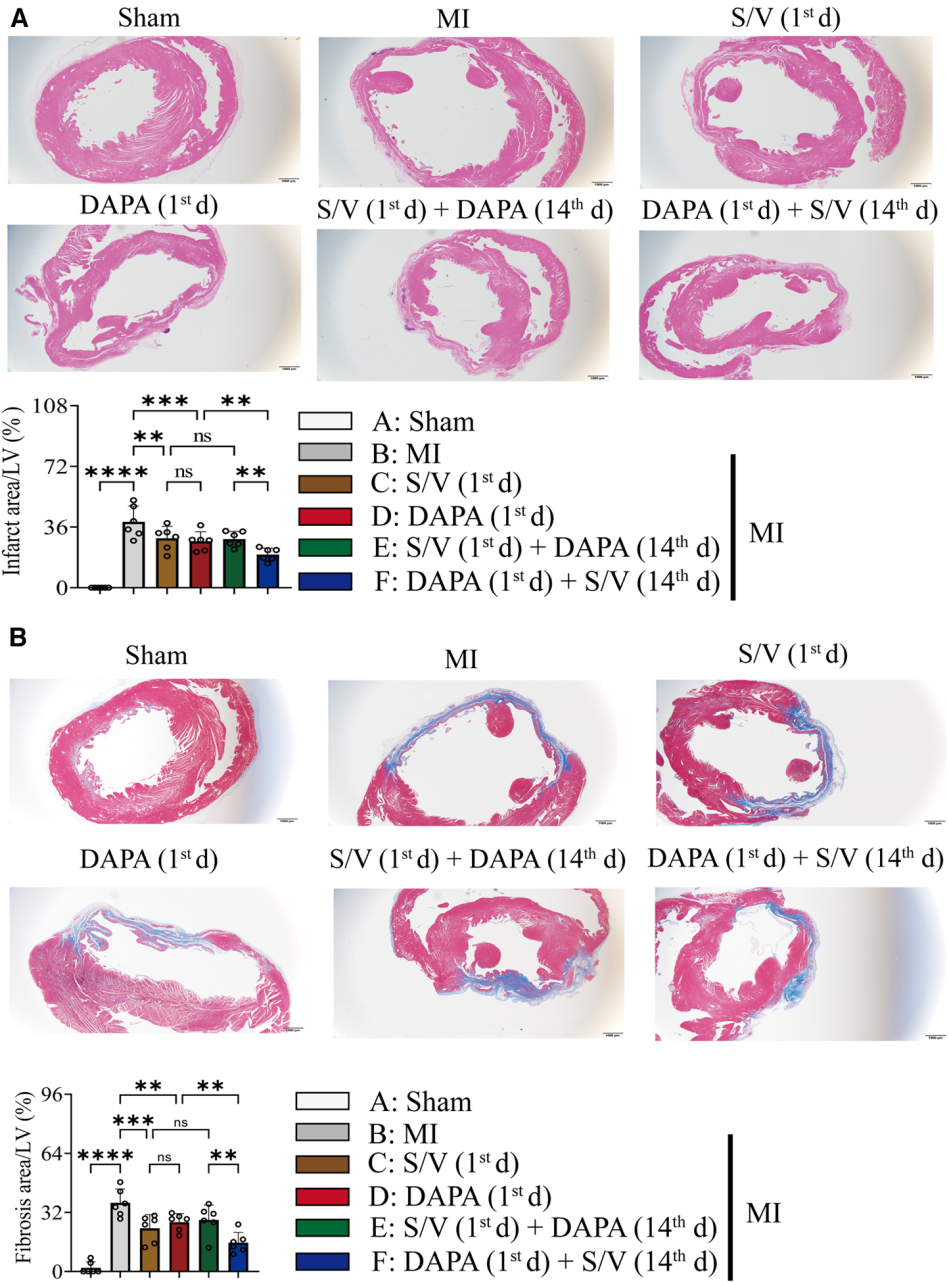
(**A**) Images and comparisons of infarction area; scale bars, 1 mm. (**B**) Images of cardiac fibrosis determined by Masson trichrome stain and comparisons of indicated parameters; scale bars, 1 mm. One-way ANOVA with Tukey's *post-hoc* test, *n* = 6 per group. *p < 0.05; **p < 0.01; ***p < 0.001; ****p < 0.0001; ns, no significance. A, Sham; B, MI; C, S/V (1st d); D, DAPA (1st d); E, S/V (1st d) + DAPA (14th d); F, DAPA (1st d) + S/V (14th d). S/V, sacubitril-valsartan; DAPA, dapagliflozin; MI, myocardial infarction; 1st, first day of dosing; 14th, day 14 dosing.

**Figure 5:**
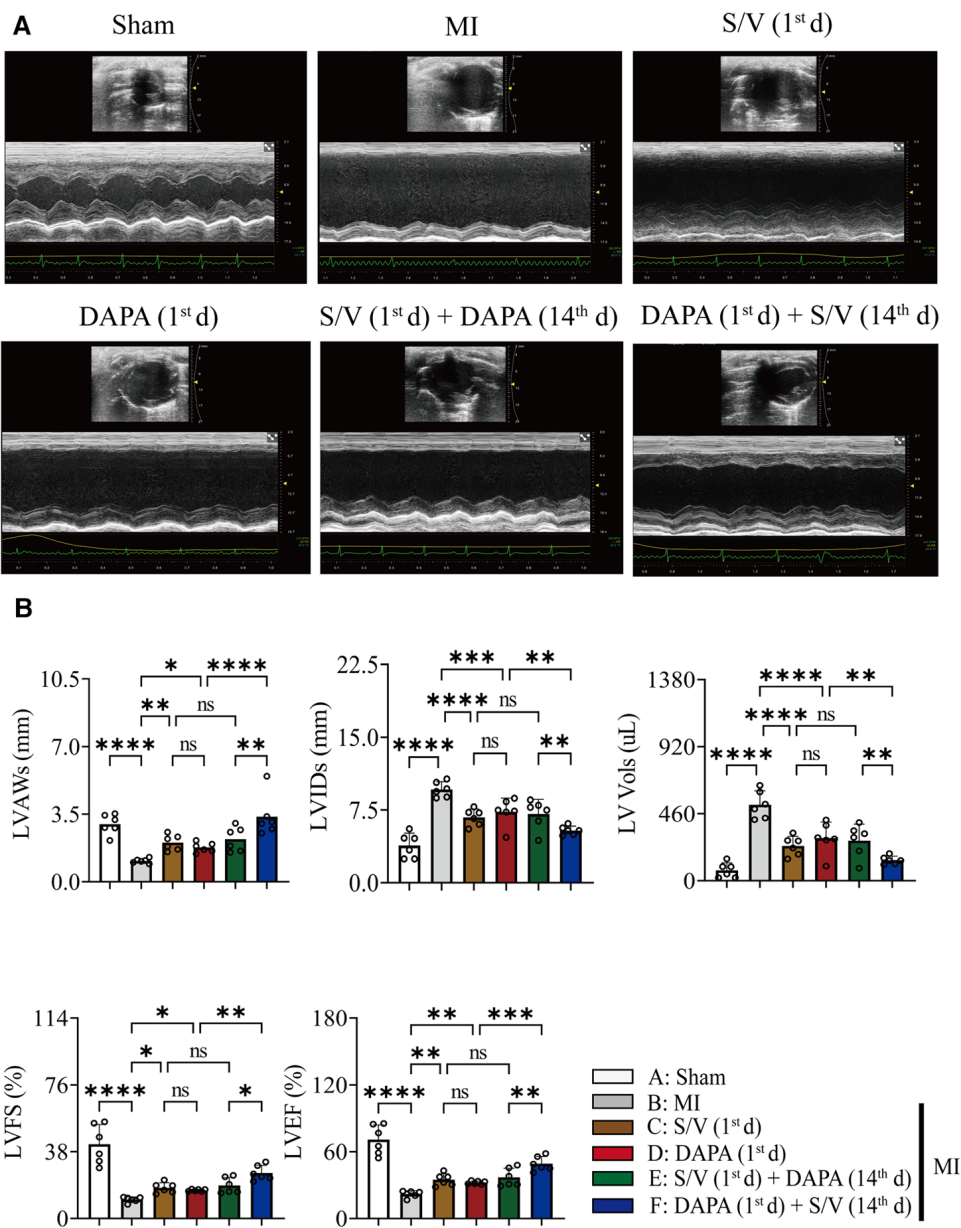
(**A**) Representative echocardiogram obtained from the mid-papillary muscle region of the LV of rats at 28 days after MI. (**B**) Echocardiography parameters 28 days after MI surgery. One-way ANOVA with Tukey's post-test, *n* = 6 per group. *p < 0.05; **p < 0.01; ***p < 0.001; ****p < 0.0001; ns, no significance. A, Sham; B, MI; C, S/V (1st d); D, DAPA (1st d); E, S/V (1st d) + DAPA (14th d); F, DAPA (1st d) + S/V (14th d). LV, left ventricular; LVAWs, left ventricular end systole anterior wall thickness; LVIDs, left ventricular end systole inner diameter; LV Vols, left ventricular end systole volume; LVFS, left ventricular fractional shortening; LVEF, left ventricular ejection fractions; S/V, sacubitril-valsartan; DAPA, dapagliflozin; MI, myocardial infarction; 1st, first day of dosing; 14th, day 14 dosing.

The related parameters analysis demonstrated that infarct and fibrosis size, and ST2 level were lowest in group A, highest in group B, and significantly lower in group F than in groups D and E, but the three groups (groups C–E) did not differ (Figures 3A, 4A,B). It was found that on the first postoperative day, the blood pressure of MI group was lower than that of the Sham group (Figure 3C). There was no significant difference in blood pressure between MI and all drug therapy groups of AMI rats (Figure 3C). Addition of S/V on the first day after AMI significantly affected blood pressure, while DAPA had a less effect on it (Figure 3C). The groups showed less interference with blood pressure but more beneficial in reducing NT-proBNP (Figure 3B). The monotherapy or the sequence of treatments [S/V (1st d) + DAPA (14th d)] limited cardiac remodeling and improved cardiac function, as reflected by higher LVAWs, LVFS, and LVEF, and lower LVIDs and LV Vols (Figures 5A,B). However, DAPA followed by S/V can better improve these ultrasound parameters in post-MI HF (Figures 5A,B).

### DAPA and/or S/V therapy could inhibit myocardial hypertrophy after experimental MI in rats

**Figure 6:**
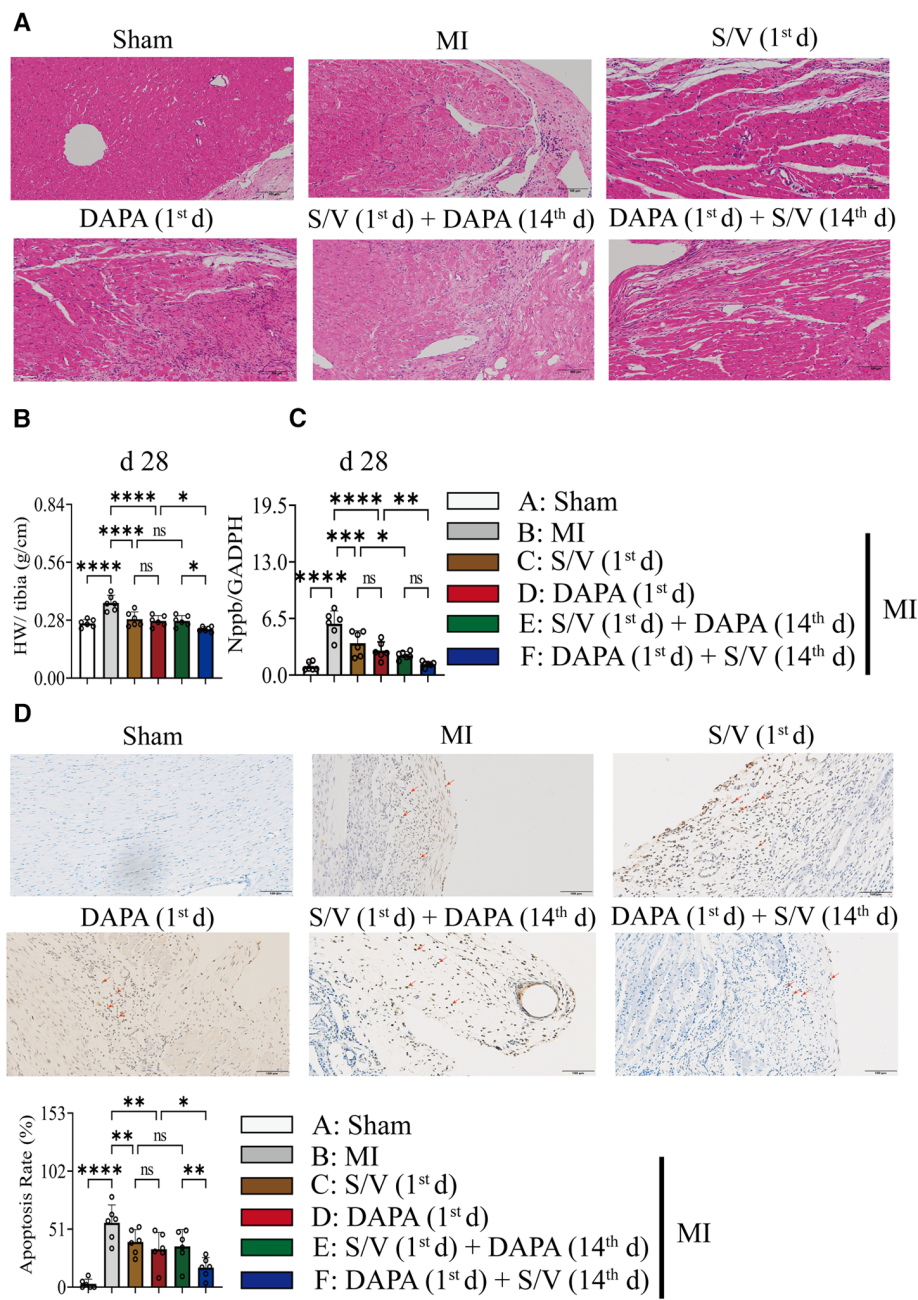
(**A**) The effect of drugs on the pathomorphology of myocardial tissue in rats (HE); scale bars, 100 µm. The observed area was infarct border. (**B**) HW to tibia length ratio. (**C**) Analysis of myocardial Nppb expression by qPCR. (**D**) TUNEL staining of apoptotic cells (red arrow) and quantitation of TUNEL staining in each group. A dark brown signal indicated positive staining; scale bars, 100 µm. One-way ANOVA with Tukey's *post-hoc* test, *n* = 6 per group. *p < 0.05; **p < 0.01; ***p < 0.001; ****p < 0.0001; ns, no significance. A, Sham; B, MI; C, S/V (1st d); D, DAPA (1st d); E, S/V (1st d) + DAPA (14th d); F, DAPA (1st d) + S/V (14th d). S/V, sacubitril-valsartan; DAPA, dapagliflozin; MI, myocardial infarction; 1st, first day of dosing; 14th, day 14 dosing; HW, heart weight; TUNEL, terminal deoxynucleotidyl transferase-mediated dUTP nick-end labeling.

One month after treatment, the heart-to-tibial length ratio was increased after MI, which could be reduced with treatments C–E (Figure 6B). Compared with groups D and E, the heart-to-tibial length ratio was further reduced in group F (Figure 6B). The results of the HE staining are shown in Figure 6A. The structure of the cardiomyocytes in the Sham operation group was normal and orderly, with no pathological changes (Figure 6A). In the MI model group, the cardiomyocyte cross-sectional area increased (Figure 6A). However, in the drug therapy groups (C–F), the area of cardiomyocyte cross-sectional was significantly reduced (Figure 6A). The mRNA level of Nppb was increased after MI, which could be reduced with treatments C–F (Figure 6C). Compared with the D group, Nppb was further reduced by group F (Figure 6C).

### DAPA and/or S/V therapy could inhibit apoptosis in myocardial after experimental MI in rats

**Figure 7:**
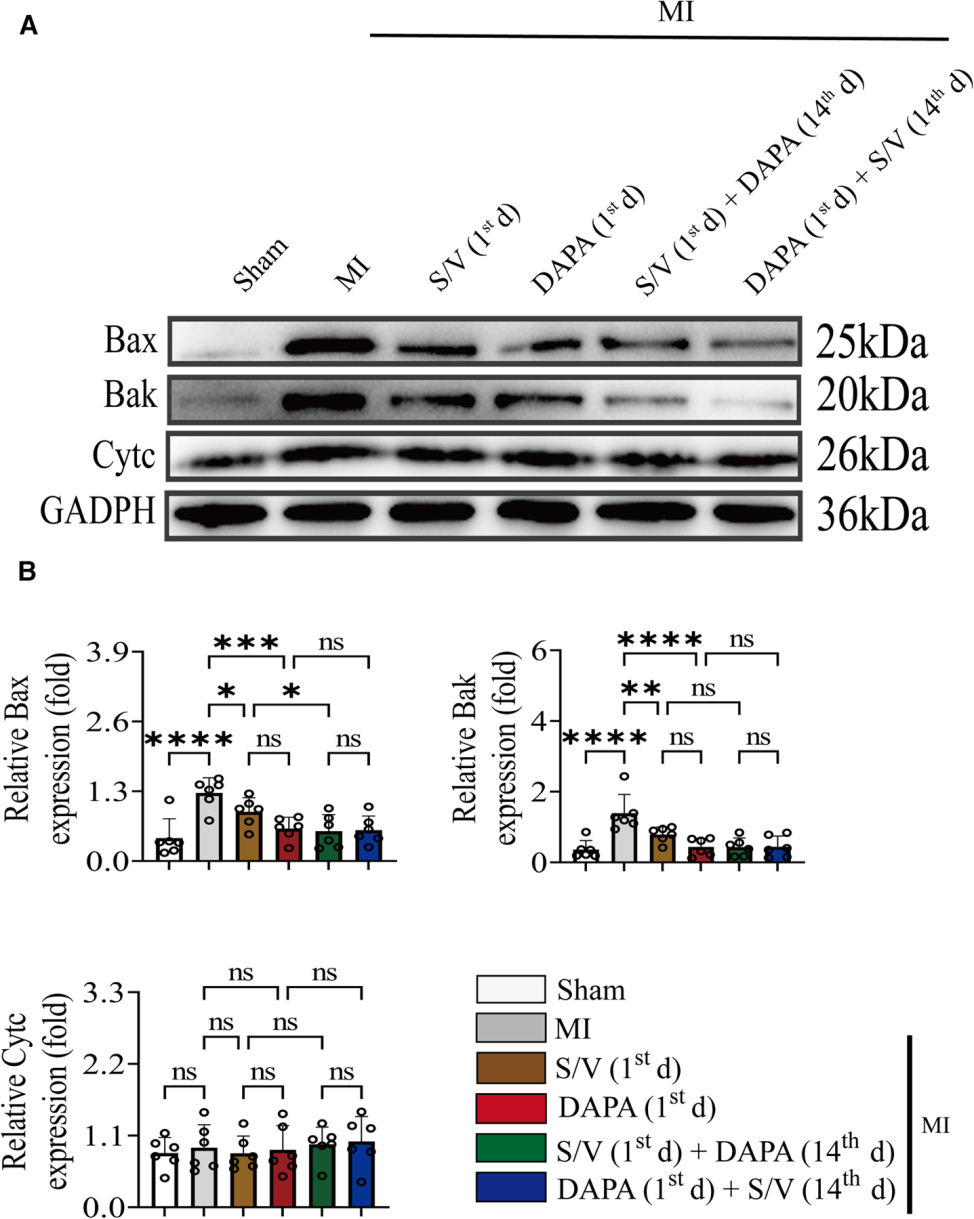
(**A,B**) Western blotting was used to assess the protein levels of Bax, Bak, and Cytc in cardiomyocytes following MI injury or drugs treatment. One-way ANOVA with Tukey's *post-hoc* test, *n* = 6 per group. *p < 0.05; **p < 0.01; ***p < 0.001; ****p < 0.0001; ns, no significance. A, Sham; B, MI; C, S/V (1st d); D, DAPA (1st d); E, S/V (1st d) + DAPA (14th d); F, DAPA (1st d) + S/V (14th d). S/V, sacubitril-valsartan; DAPA, dapagliflozin; MI, myocardial infarction; 1st, first day of dosing; 14th, day 14 dosing.

**Figure 8:**
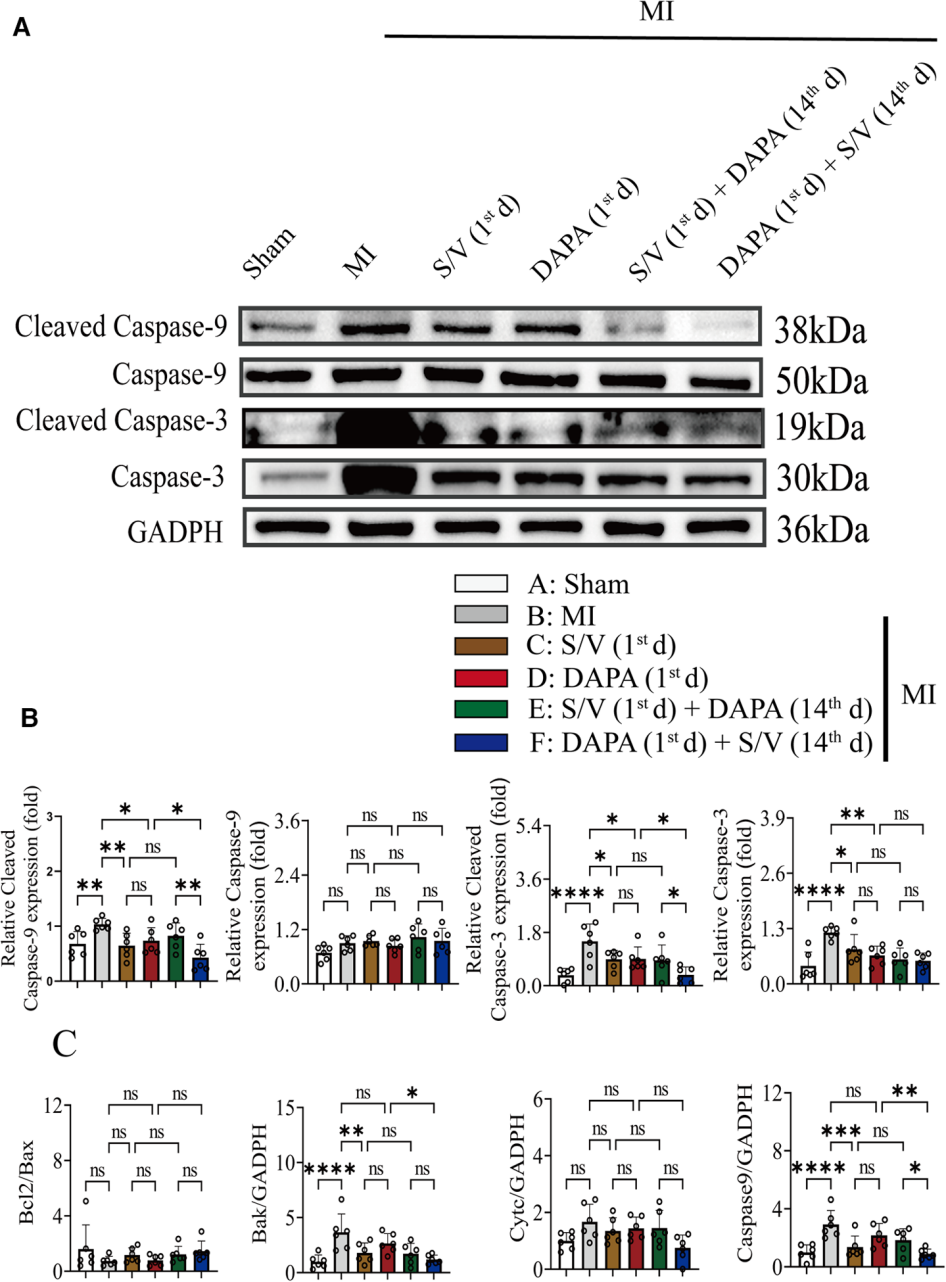
(**A,B**) Western blotting was used to assess the protein levels of Cleave Caspase-9, Caspase-9, Cleaved Caspase-3, and Caspase-3 in cardiomyocytes following MI injury or drugs treatment. (**C**). Analysis of myocardial Bcl2/Bax, Bak, Cytc, and Caspase-7 expression by qPCR. One-way ANOVA with Tukey's *post-hoc* test, *n* = 6 per group. *p < 0.05; **p < 0.01; ***p < 0.001; ****p < 0.0001; ns, no significance. A, Sham; B, MI; C, S/V (1st d); D, DAPA (1st d); E, S/V (1st d) + DAPA (14th d); F, DAPA (1st d) + S/V (14th d). S/V, sacubitril-valsartan; DAPA, dapagliflozin; MI, myocardial infarction; 1st, first day of dosing; 14th, day 14 dosing.

Results of TUNEL staining showed that the apoptosis rate of myocardia stimulated with MI was markedly increased, and significantly lowered in group F than in groups D and E. There was no significant difference in the three groups (C–E) (Figure 6D). The protein levels of Bax, Bak, and Caspase-3 can be significantly reduced by DAPA and/or S/V (Figures 7A,B, 8A,B). One month after treatment, the protein levels of Cleaved Caspase-9 and Cleaved Caspase-3 and the mRNA levels of Caspase-9 were increased after MI, which could be reduced with treatments C–E (Figures 8A–C). Compared with D and E groups, the protein levels of Cleaved Caspase-9 and Cleaved Caspase-3 and the mRNA levels of Caspase-9 were further reduced by group F (Figures 8A–C). Additionally, DAPA and/or S/V were also associated with lower protein and mRNA levels of Bak (Figures 7A,B, 8C). The protein levels of Cytc and Caspase-9, as well as the mRNA levels of Bcl2/Bax and Cytc, were similar among the groups (Figures 7A,B, 8A–C).

### DAPA and/or S/V therapy could suppress oxidative stress and fibrosis signaling after experimental MI in rats

**Figure 9:**
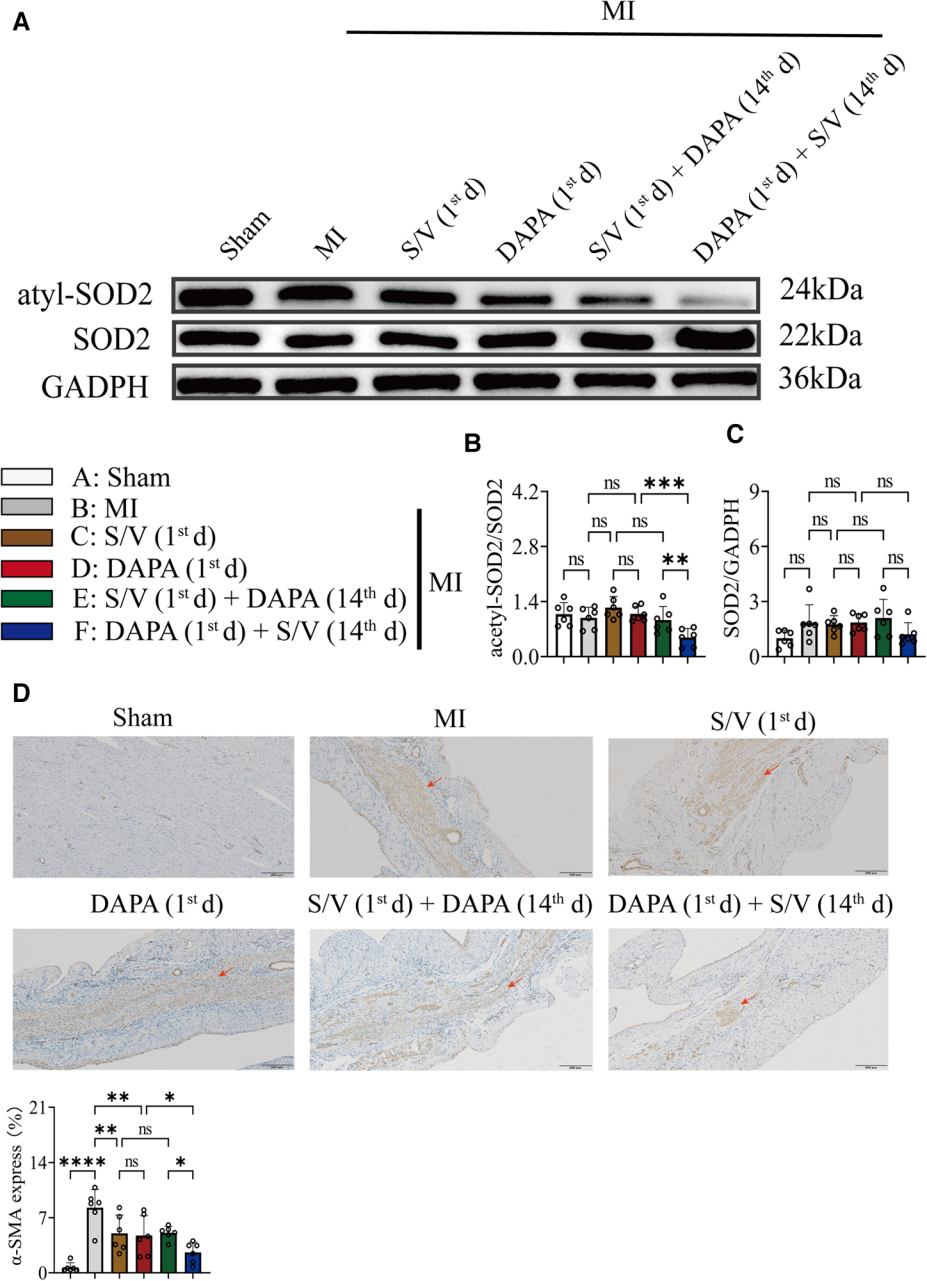
(**A,B**) Western blotting was used to assess the protein levels of acetyl-SOD2 and SOD2 in cardiomyocytes following MI injury or drugs treatment. (**C**) Analysis of myocardial SOD2 expression by qPCR. (**D**). Immunohistochemistry of myocardial tissue showed that expression of α-SMA and quantitation of α-SMA in each group; scale bars, 200 µm. The observed area was infarcted region (a dark brown signal indicates α-SMA-positive area). One-way ANOVA with Tukey's *post-hoc* test, *n* = 6 per group. *p < 0.05; **p < 0.01; ***p < 0.001; ****p < 0.0001; ns, no significance. A, Sham; B, MI; C, S/V (1st d); D, DAPA (1st d); E, S/V (1st d) + DAPA (14th d); F, DAPA (1st d) + S/V (14th d). S/V, sacubitril-valsartan; DAPA, dapagliflozin; MI, myocardial infarction; 1st, first day of dosing; 14th, day 14 dosing; α-SMA, alpha-smooth muscle actin.

**Figure 10:**
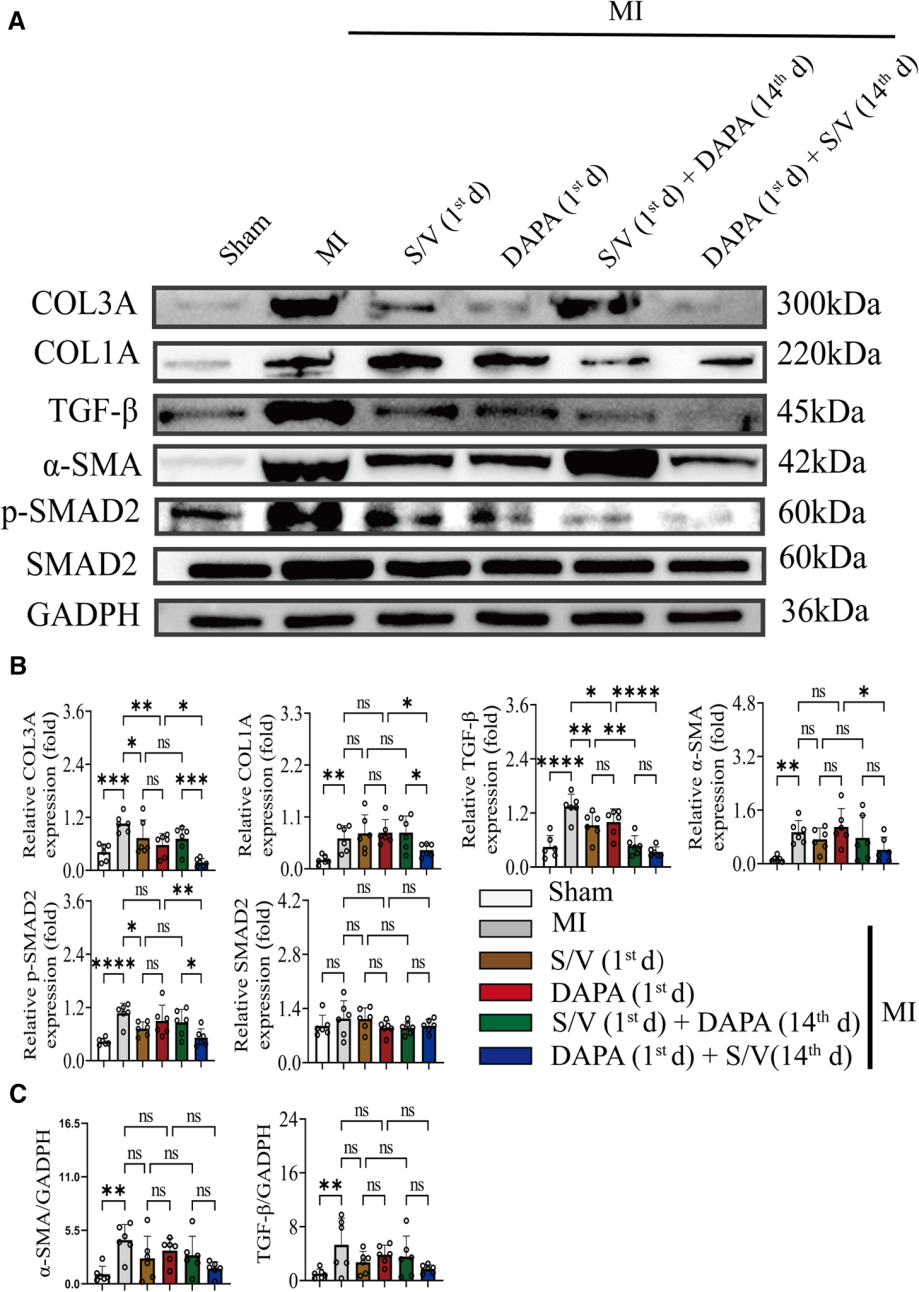
(**A,B**) Western blotting was used to assess the protein levels of COL3A, COL1A, α-SMA, TGF-β, SMAD2, and p-SMAD2 in cardiomyocytes following MI injury or drugs treatment. (**C**) Analysis of myocardial α-SMA and TGF-β expression by qPCR. One-way ANOVA with Tukey's *post-hoc* test, *n* = 6 per group. *p < 0.05; **p < 0.01; ***p < 0.001; ****p < 0.0001; ns, no significance. A, Sham; B, MI; C, S/V (1st d); D, DAPA (1st d); E, S/V (1st d) + DAPA (14th d); F, DAPA (1st d) + S/V (14th d). S/V, sacubitril-valsartan; DAPA, dapagliflozin; MI, myocardial infarction; 1st, first day of dosing; 14th, day 14 dosing; α-SMA, alpha-smooth muscle actin.

In addition, Western blotting analysis of protein extraction with an acetyl-SOD2 antibody was suppressed by group F than other groups (Figures 9A,B). The mRNA level of SOD2 was similar among the groups (Figure 9C). Immunohistochemistry of myocardial tissue showed that expression of α-SMA was decreased after groups C–E treatment and significantly abridged after group F therapy, and there were no obvious differences after groups C–E (Figure 9D). The Western blotting analysis demonstrated that COL3A and p-SMAD2 were lowest in group A, highest in group B, and significantly lower in group F than in groups D and E, and there was no significant difference in the three groups (C–E) (Figures 10A,B). The protein level of TGF-β was significantly decreased in group E and F than in the MI and monotherapy groups (Figures 10A,B). The protein expression of COL1A had improvement in group F compared to the other treatment groups (Figures 10A,B). The protein levels of SMAD2 and α-SMA, as well as the mRNA levels of α-SMA and TGF-β, were similar among the treatment groups (Figures 10A–C).

### DAPA therapy could suppress inflammation signaling and activate oxidative phosphorylation after experimental MI in rats

To systematically investigate the mechanism of cardiac protection from DAPA, the normal myocardium (group Sham) and infarct myocardium of the MI (group MI) and MI + DAPA (1st d) were analyzed accordingly by RNA-seq technology (GSE229147, https://www.ncbi.nlm.nih.gov/geo/query/acc.cgi?acc=GSE229147). DEGs between each two groups were screened with the criteria of a fold change ≥2 (log_2_FC > 1) and *p*-value <0.05.

**Figure 11:**
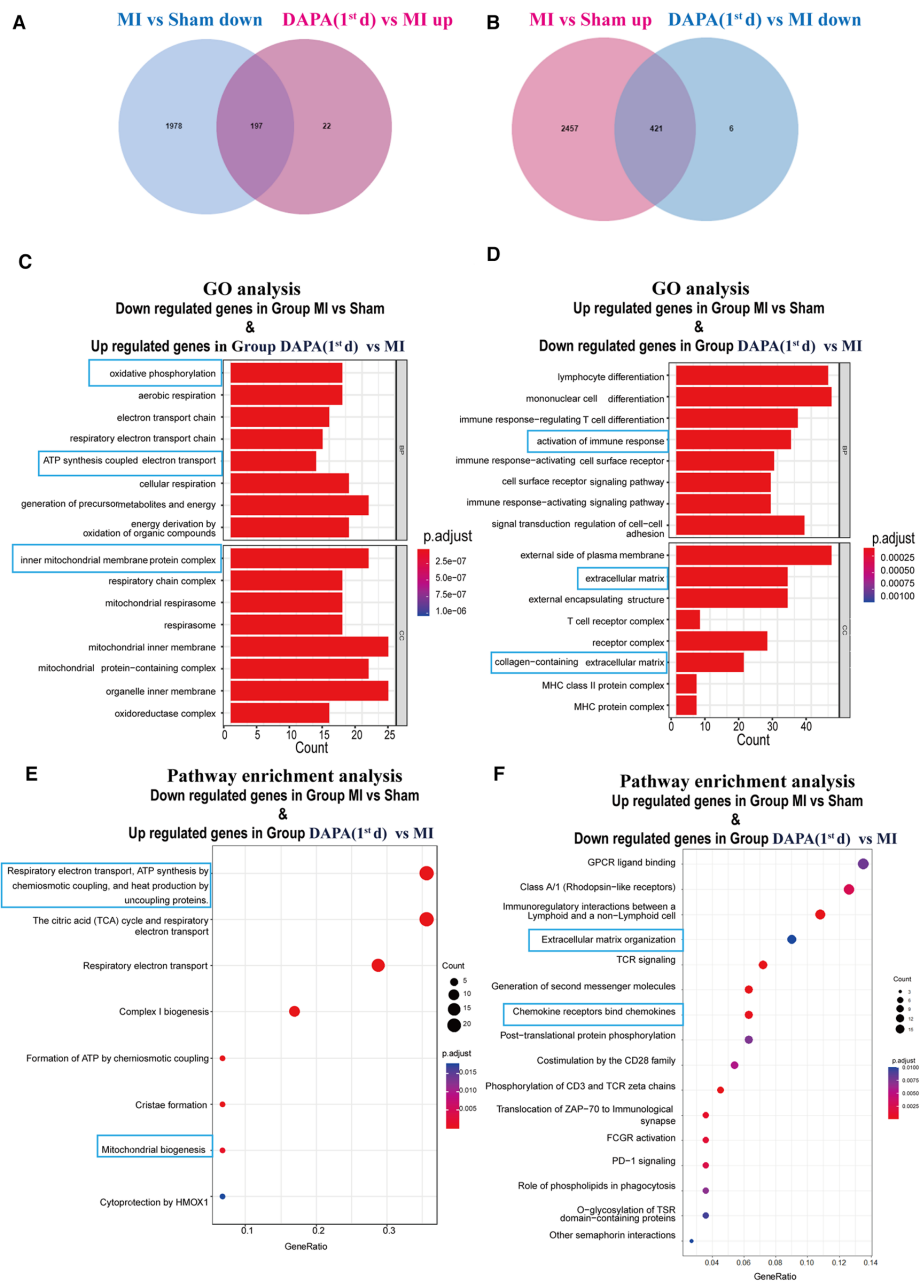
DAPA exerts a protective effect after MI mainly through activation of OXPHOS. (**A**) DEGs overlapping between genes which are downregulated in group MI vs. group Sham and genes which are upregulated in group DAPA (1st d) vs. group MI. (**B**) DEGs overlapping between genes which are upregulated in group MI vs. group Sham and genes which are downregulated in group DAPA(1st d) vs. group MI. (**C,E**) Bar chart and bubble chart, respectively, show GO (BP, biological processes; CC, cellular components) and Reactome pathways enriched in DEGs overlapping between genes which are downregulated in group MI vs. group Sham and genes which are upregulated in group DAPA(1st d) vs. group MI. (**D,F**) Bar chart and bubble chart, respectively, show GO and Reactome pathways enriched in DEGs overlapping between genes which are upregulated in group MI vs. group Sham and genes which are downregulated in group DAPA(1st d) vs. group MI. DAPA, dapagliflozin; MI, myocardial infarction; 1st, first day of dosing; OXPHOS, oxidative phosphorylation; DEGs, differentially expressed genes; GO, Gene Ontology.

To find the possible benefits of DAPA, downregulated genes in group MI vs. group Sham, which potentially maintained normal function of cardiomyocyte, were picked up to determine the overlap with upregulated genes in group MI + DAPA (1st d) vs. group MI. According to our analyses, 2,175 genes were significantly downregulated in infarct myocardium, and 197 genes among them were significantly rescued by DAPA treatment (Figure 11A), whereas upregulated genes in group MI vs. group Sham, as the risk genes of post-MI HF, were picked up to determine the overlap with downregulated genes in group MI + DAPA (1st d) vs. group MI. A total of 2,878 genes were significantly upregulated in infarct myocardium, and 421 genes among them were significantly rescued by DAPA treatment (Figure 11B).

To identify the key pathways reversed by DAPA treatment in MI, we further performed GO and pathway analyses. GO analysis suggested that biological processes (BP) such as oxidative phosphorylation (OXPHOS) and adenosine triphosphate (ATP) synthesis coupled electron transport, as well as the mitochondrial inner membrane as cellular components (CC) were strongly enhanced by DAPA treatment in MI (Figure 11C). Furthermore, activation of immune response and collagen-containing extracellular were attenuated by DAPA, in accord with the improvement of inflammation and fibrosis under DAPA treatment (Figure 11D).

**Figure 12:**
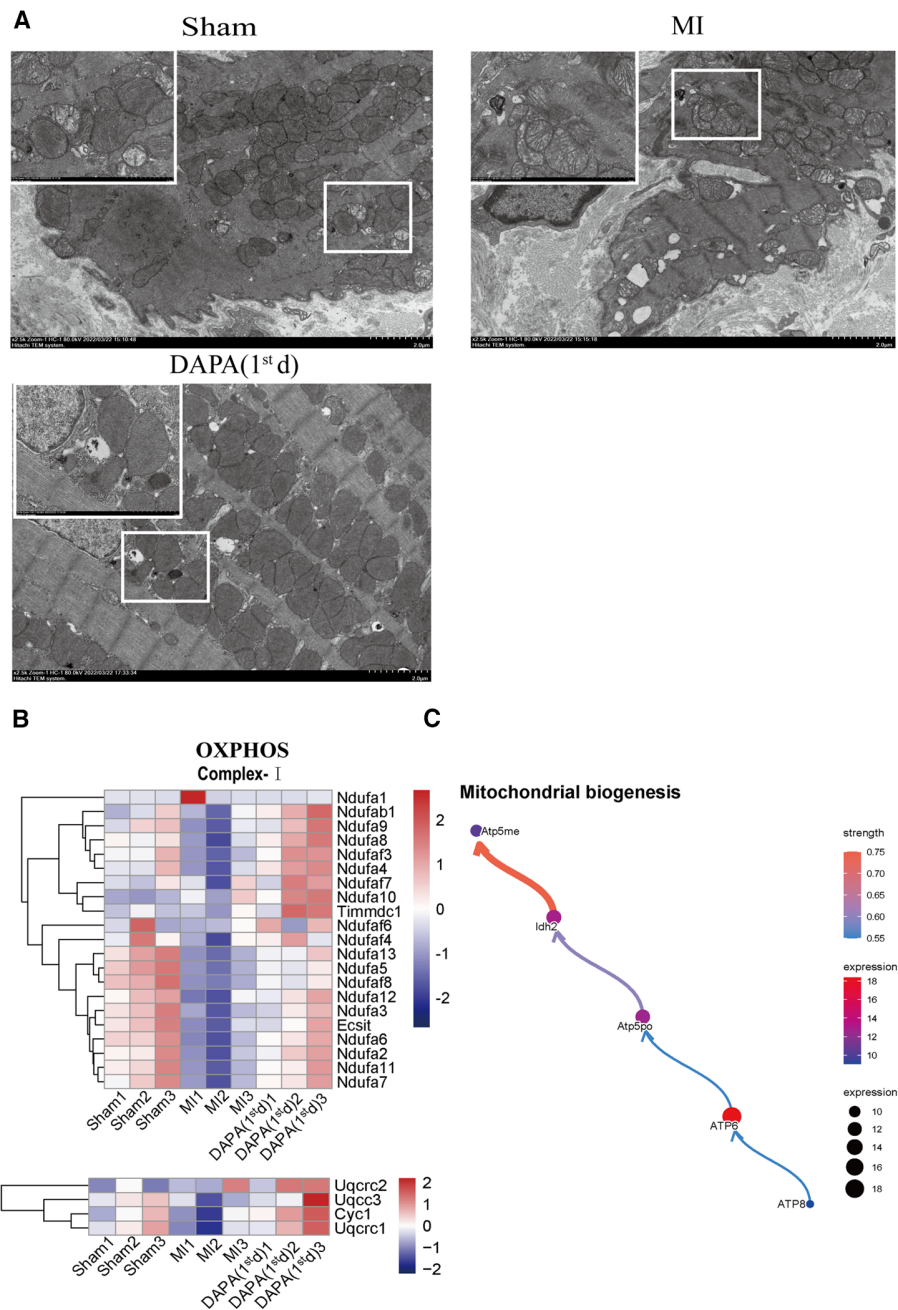
DAPA upregulates mitochondrial biogenesis and improves OXPHOS complexes mitochondrial integrity. (**A**) Transmission electron micrograph of the myocardium; scale bars, 2 or 1 μm. The observed area was infarct border. (**B**) Relative transcriptional abundance of mitochondrial OXPHOS Complex subunits in normal myocardium (group Sham), infarct myocardium of the MI (group MI) vs. group DAPA (1st d). (**C**) CBNplot shows relationships between mitochondrial biogenesis related genes. DAPA, dapagliflozin; MI, myocardial infarction; 1st, first day of dosing; OXPHOS, oxidative phosphorylation.

By visualizing the clustered KEGG pathway results, we found that pathways related to the citric acid (TCA) cycle and respiratory electron transport and mitochondrial biogenesis were significantly upregulated by DAPA (Figure 11E), while pathways related to inflammation and fibrosis were downregulated by DAPA in MI (Figure 11F). During MI, mitochondria are central to mediating the damage that underlies myocardial injury (19). In adult cardiomyocytes, mitochondria are the major cellular powerhouse and produce over 95% of the cell's energy in the form of ATP (20). Energy in the form of ATP is generated mainly in mitochondria by the OXPHOS process, in which electrons produced by the TCA cycle are transferred down the mitochondrial respiratory complexes (21). Mitochondria have evolved to control a diverse number of processes including cellular energy production, inflammation, fibrosis, and apoptosis (19, 22–24). During myocardial injury, multiple signaling pathways can stimulate mitochondria to increase ATP production to compensate for the rapid loss of energy (25). A transmission electron microscope (TEM) was used to observe the morphological features of mitochondria. In the sham group, these mitochondria were generally seen individually and appeared to have intact membranes and cristae (Figure 12A). Ultrastructural alterations of the mitochondria observed in the MI group were as follows: extensive mitochondrial cristae breakdown, highly swollen mitochondria that had lost all their cristae, and rupture of the mitochondrial membrane (Figure 12A), but DAPA treatment had significantly changed these alterations (Figure 12A).

Therefore, gene expression profiles related to OXPHOS complex subunits among the normal myocardium (group Sham) and infarct myocardium of the MI (group MI) and MI + group DAPA (1st d) were extracted and analyzed from RNA-seq data (Figure 12B). The results showed the expression of majority subunits in Complex I (Ndufa7, Ndufa11, Ndufaf2, Ndufaf6, Exsit, etc.) and Complex III (Uqcrc1, Cyc 1, Uqcc3, Uqcrc2) were significantly upregulated in DAPA treatment [group DAPA (1st d)] under normal physiological conditions. In addition, we describe the relationship between mitochondrial biogenesis related genes by CBNplot (Figure 12C). Above all, we conclude that DAPA may bring improvement in cardiac function by enhance myocardial mitochondrial biogenesis and OXPHOS, which protect myocardium from inflammation and fibrosis.

## Discussion

We evaluated the effect of DAPA and/or S/V in post-MI HF and found substantial preclinical implications. First, we found no considerable difference in the cardioprotective effects between singular DAPA or S/V in rats with post-MI HF. Second, the most effective treatment strategy for rats with post-MI HF is the administration of DAPA during the first 2 weeks, followed by the addition of S/V to DAPA later. Moreover, adopting a therapeutic scheme whereby S/V was administered first, followed by later addition of DAPA, failed to further improve cardiac function compared to S/V monotherapy.

S/V effectively ameliorates symptoms in patients with chronic HF and AMI (6, 7, 26). In rats, S/V results in a marked improvement in cardiac function and reductions in oxidative stress and inflammatory processes following MI (27). Large clinical studies have shown that SGLT2i decreases in the incidence of cardiovascular deaths and HF hospitalizations in patients with chronic HF (28). In a murine model of cardiac hypertrophy, SGLT2i reduces LV fibrosis via increased AMPK signaling (29). In AMI rats with or without diabetes mellitus, SGLT2i exerts cardioprotective effects by modulating autophagic flux (30). Despite a growing number of investigations, the role and impact of SGLT2i in AMI therapy are largely unclear. Few studies have compared the efficacy of S/V and DAPA alone and in combination in post-MI patients with HF. A correlational investigation revealed that patients with HFrEF who received combination therapy with DAPA and S/V demonstrated notably enhanced cardiovascular benefits in comparison to those in individuals receiving monotherapy with S/V (31). In addition, the effects of the co-administration of S/V and DAPA surpassed those of monotherapy in protecting the rat myocardium against ischemia-reperfusion (I/R) injury (32). Based on the results of these studies, we used an animal model of MI to further investigate the effects of the early initiation of DAPA or different orders of administration in combination therapy with S/V on heart function in post-MI HF. Our model simulates severe AMI, typically characterized by a large infarct size and hypotension. By utilizing this model, we can gain a greater comprehension of the intricacies underlying this disorder and screening more efficacious interventions. Our findings indicated that the concurrent administration of DAPA and S/V was not a suitable approach, within 3 days following the onset of AMI. The diuretic properties of both DAPA and SV prompted hypotension (data not shown) and consequent hemodynamic instability in post-AMI. S/V inhibited the breakdown of natriuretic peptide, thereby promoting natriuresis and diuresis (33). DAPA also induces natriuresis and glucose osmotic diuresis by inhibiting SGLT2 in the renal proximal tubule (34). Consequently, in subsequent experiments, the group treated with the combination of DAPA and S/V within one day of AMI was reasonably excluded.

Initially, a comparison of the singular administrations of DAPA or S/V was conducted to assess their individual effectiveness and safety in post-MI HF. Our study indicated that both DAPA and S/V substantially improved cardiac structure and function in rats with post-MI HF. In the DAPA or S/V monotherapy setting, comparable reductions in infarct size, as well as myocardial hypertrophy, apoptosis, and fibrosis were observed. Our findings suggest that DAPA and S/V confer similar cardioprotective effects in post-MI HF. Moreover, we investigated the sequence of administration of DAPA and S/V. Our results revealed that DAPA followed by S/V exhibited the highest efficacy in preventing post-MI HF in rats. We hypothesized that the substantial improvement in heart function obtained by DAPA followed by S/V could be attributed to the prompt initiation of DAPA, specifically within 24 h of AMI. To gain further insights into the mechanisms underlying the cardioprotective effects of DAPA in AMI, we conducted RNA-seq. Our RNA-Seq data revealed that DAPA treatment after AMI altered the expression of genes related to myocardial mitochondrial biogenesis and OXPHOS. Mitochondria play a pivotal role in mediating the damage associated with myocardial injury during MI (19, 35). Within the context of HF, mitochondrial dysfunction can trigger the cytosolic buildup of deleterious glucose and lipid metabolites, instigating a progressive decline in ATP production and accelerating the pathogenesis of HF (36).

SGLT2i exerts its beneficial effects on cardiomyocyte energy metabolism via a trio of complementary mechanisms. SGLT2i elicits a metabolic state resembling starvation, which is characterized by the urinary glucose excretion, the induction of ketogenesis, and the reduction of adipose tissue depots at a systemic level (36, 37). Furthermore, SGLT2i augments autophagic flux, which facilitates the clearance of dysfunctional mitochondria as well as toxic glucose and lipid by-products (38–40). SGLT2i directly interacts with GLUT1 and GLUT4, leading to modifications in their respective activities. This has the potential to augment myocardial glucose and fatty acids oxidation (41, 42). The processes observed within cardiomyocytes are concomitant with a rise in expression of sensors that respond to nutrient deprivation and a reduction in the expression of sensors that respond to nutrient surplus. These changes serve to support mitochondrial biogenesis and augment mitochondrial OXPHOS, thereby promoting ATP production (43, 44) In light of the substantial loss of mitochondria (45), our results indicate that DAPA failed to confer added benefits to rat cardiac function beyond 2 weeks of AMI. Hence, prompt administration of DAPA should be considered, as its impact on blood pressure levels following AMI was minimal.

Our results demonstrated that S/V can enhance cardiac function following AMI. S/V is a composite consisting of sacubitril (a neprilysin inhibitor) and valsartan. By inhibiting AT1 receptors on vascular and adrenal cells (46), valsartan has the potential to decrease cardiac hypertrophy, inflammation and fibrosis (47). In addition to suppressing the RAAS and sympathetic systems (47), natriuretic peptides reduce cardiac hypertrophy, inflammation, apoptosis, and fibrosis (48–51), while facilitating sodium excretion and diuresis (52). Moreover, the activation of neurohormonal pathways and hemodynamic perturbations observed in both animals and humans following AMI may impair cardiac contractility, resulting in systemic hypotension and inadequate organ perfusion (53–55). As such, S/V exhibited a more pronounced effect on blood pressure during the initial phase of AMI, whereas no notable alterations were evident after a 2-week period. From a clinical standpoint, S/V therapy is constrained in certain patients with AMI exhibiting hypotension and may be limited by the need for a gradual titration process to attain the desired dosage or the maximum tolerable level for an individual patient. In the case of SGLT2i therapy, the initial dosage corresponds with the intended target treatment level.

Together, we concluded that DAPA can be used preferentially in post-MI HF. After 2 weeks of treatment with DAPA, the addition of S/V could be an optimal strategy, substantially improving heart function in rats with post-MI HF. Although these findings are promising from a fundamental research perspective, further clinical studies are required to substantiate this treatment approach. More clinical research is needed to verify that SGLT2i improves outcomes in patients with AMI. Furthermore, additional comparative analyses of S/V and DAPA as well as their combined effects in patients with AMI are needed.

### Study limitation

Owing to the extensive infarct size in our model, a combination of DAPA and S/V at 24 h post-AMI was deemed unfeasible. Subsequently, we intend to exploit the I/R model to determine the feasibility of combining DAPA and S/V following a 24-hour period. Furthermore, rather than titrating S/V, we administered a substantial dose, leading to a reduction in blood pressure in rats with MI. Moreover, the degree to which SGLT2i and S/V impact cardiac remodeling in the presence of conventional post-MI HF therapy remains unknown.

## Conclusions

Our study revealed no notable difference in the cardioprotective effects of singular DAPA or S/V treatment in rats with post-MI HF. The most effective treatment strategy for rats with post-MI HF was the administration of DAPA during the first 2 weeks, followed by the addition of S/V to DAPA later. Conversely, adopting a therapeutic scheme whereby S/V was administered first, followed by later addition of DAPA, failed to further improve cardiac function compared to S/V monotherapy.

## Data Availability

The original contributions presented in the study are publicly available. This data can be found here: https://www.ncbi.nlm.nih.gov/geo/query/acc.cgi?acc=GSE229147.
